# Long-term clinical control in chronic rhinosinusitis: Outcomes more than five years after surgery

**DOI:** 10.1007/s00405-025-09529-z

**Published:** 2025-08-09

**Authors:** Gonneke E. Joustra, Marc C. den Heijer, Rahma Q. H. al Yousef, Karin M. Vermeulen, György B. Halmos, Astrid G. W. Korsten-Meijer, Robert A. Feijen

**Affiliations:** 1 Department of Otorhinolaryngology – Head and Neck Surgery, University Medical Center Groningen, University of Groningen, Groningen, The Netherlands; 2 Graduate School of Medical Sciences, University of Groningen, Groningen, The Netherlands; 3 Department of Epidemiology, University Medical Center Groningen, University of Groningen, Groningen, The Netherlands

**Keywords:** EES-Q, Chronic rhinosinusitis, Quality of life, Patient-reported outcome measure, Clinical control, Disease control

## Abstract

**Purpose:**

Chronic rhinosinusitis (CRS) is a common chronic and disabling disease with a high socioeconomic burden. The primary goal of treatment is improving health-related quality of life (HRQoL) and maintaining clinical control. The aim of this study was to assess clinical control in CRS patients more than 5 years after endoscopic endonasal surgery (EES).

**Methods:**

In this observational cohort study, 123 patients with CRS were included and they completed the Endoscopic Endonasal Sinus and Skull Base Surgery Questionnaire and updated European Position Paper on Rhinosinusitis and Nasal Polyps (EPOS) criteria. Three clinical control groups were obtained according to the EPOS criteria and corresponding EES-Q and domain scores were analyzed. Univariate analyses were performed to identify variables significantly associated with outcome.

**Results:**

Symptoms of 25.2% of CRS patients were ‘controlled’, 26.8% were ‘partly controlled’ and 48.0% were ‘uncontrolled’ according to the EPOS criteria. Nonsteroidal anti-inflammatory drug – exacerbated respiratory disease, allergic rhinitis, revision surgery, smoking, asthma, and nasal polyps were all significantly associated with partly controlled or uncontrolled CRS. The physical domain played the most significant role in disease control, followed by the social and psychological domain.

**Conclusion:**

According to the updated EPOS criteria, only 25% of patients had controlled CRS more than five years following EES. This emphasizes the importance of unravelling the factors that contribute to disease control to improve treatment. The multidimensional aspect should be taken into account as well as the patients’ perspective.

Level of evidence: IIB

**Supplementary Information:**

The online version contains supplementary material available at 10.1007/s00405-025-09529-z.

## Introduction

Affecting around 11% of the total European population, chronic rhinosinusitis (CRS) is a common chronic and disabling disease with a high socioeconomic burden [1–4]. The primary goal of treatment of chronic rhinosinusitis (CRS) is improving the patients’ health-related quality of life (HRQoL) and maintaining clinical control [2, 5]. Clinical control is defined as a ‘disease state with acceptable levels of disease manifestation’ [2, 5]. CRS physically affects patients, but can also have a high social and psychological burden [5–9]. Although there are several treatment options to alleviate symptoms some patients fail to achieve clinical control.

In 2012 the European Position Paper on Rhinosinusitis and Nasal Polyps (EPOS) introduced a concept of assessing clinical control of CRS [10] and following validation studies on its clinical applicability [11–13], an updated staging system was published in 2020 (Table 1) [2]. Three subgroups could be obtained: controlled, partly controlled and uncontrolled CRS [2]. However, these criteria have not been validated yet. In addition, the underlying issues contributing to a suboptimal disease control were not sufficiently captured with only the EPOS criteria. Therefore, the Endoscopic Endonasal Sinus and Skull Base Surgery Questionnaire (EES-Q, appendix) will be used. A questionnaire designed to evaluate multidimensional HRQoL after endoscopic endonasal surgery (EES) [14–16]. Recently, the EES-Q has specifically been validated for CRS patients with [16] and without surgery [17].

**Table 1:** Assessment of current clinical control of CRS (in the last month), EPOS update 2020 [2]

	Controlled (all of the following)	Partly controlled (at least 1 present)	Uncontrolled (3 or more present)
**Nasal blockage**^1^	Not present or not bothersome^2^	Present on most days of the week^3^	Present on most days of the week^3^
**Rhinorrhea/Postnasal drip**^1^	Little and mucous^2^	Mucopurulent on most days of the week^3^	Mucopurulent on most days of the week^3^
**Facial pain/Pressure**^1^	Not present or not bothersome^2^	Present on most days of the week^3^	Present on most days of the week^3^
**Smell**^1^	Normal or only slightly impaired^2^	Impaired^3^	Impaired^3^
**Sleep disturbance or fatigue**^1^	Not present^2^	Present^3^	Present^3^
**Nasal endoscopy** (if available)	Healthy or almost healthy mucosa	Diseased mucosa^4^	Diseased mucosa^4^
**Rescue treatment** (in last 6 months)	Not needed	Need of 1 course of rescue treatment	Symptoms (as above) persist despite rescue treatment(s)

^1^ Symptoms of CRS; ^2^ For research VAS ≤ 5; ^3^ For research VAS > 5; ^4^ Showing nasal polyps, mucopurulent secretions or inflamed mucosa

Therefore, the aim of this study was to assess clinical control criteria in CRS patients more than 5 years after EES using the EES-Q and the updated EPOS criteria, and evaluate their true benefits and how they relate to each other. Furthermore, the goal was to obtain better insight in the level of control of CRS and unravel the factors that contribute to a partly or uncontrolled level of CRS to improve individualized treatment.

## Methods

This observational cohort study was conducted at the Department of Otorhinolaryngology – Head and Neck Surgery in a tertiary referral center. Local institutional review board approval was obtained before commencing (RR 202000102).

### Study population

Between May and June 2021, a convenience sample of 165 CRS patients—who had undergone EES 6 to 8 years earlier and had completed the EES-Q up to one year postoperatively—were consecutively invited to participate in the study. No selection criteria were applied, and all who agreed to participate were included. All patients had CRS defined by the EPOS criteria [2] and were aged ≥ 18 years and able to read and write Dutch. Seven patients did not have an email address, 35 patients did not complete the questionnaire. The remaining 123 patients returned the completed the questionnaires digitally and were included for analyses. Written information was provided and informed consent was obtained. Relevant patient characteristics were extracted from their electronic medical record to ensure accuracy and consistency of the data. All patients received standard postoperative treatment with saline rinsing and an intranasal corticosteroid nasal spray on a daily basis.

### EPOS staging system

The updated EPOS staging system (Table 1) covers nasal blockage, rhinorrhea or postnasal drip, facial pain or pressure, smell and sleep disturbance or fatigue [2]. In our study, patients were asked to indicate to what extent they experienced a blocked nose, a runny nose or postnasal drip, facial pain or pressure, a reduced sense of smell or sleep disturbance or fatigue. Symptoms were scored in relation to CRS over the past month using a visual analogue scale (VAS) to indicate their degree of inconvenience, ranging from not bothersome (1) to extremely bothersome (10) [2]. Additionally, the need for rescue treatment in the last 6 months was assessed. In our study no nasal endoscopy was performed at the time of completing the questionnaire. Based on the outcomes, CRS patients were divided into ‘controlled’, ‘partly controlled’ or ‘uncontrolled’ groups [2].

### Endoscopic endonasal sinus and skull base surgery questionnaire

The EES-Q (appendix) is a validated, patient-reported outcome questionnaire (30 items) including physical, psychological and social domains [15]. A five-point Likert response scale, ranging from not at all (1), to very severely (5) is used to indicate the degree of inconvenience. Lower scores indicate better HRQoL. Completion typically requires 3–5 min. Domain scores were calculated to create an easily interpretable score, ranging from 0 (not bothered at all) to 100 (very severely bothered). Domain scores were calculated by summing the 10-item score of each domain, subtracting 10 points from this total and multiplying this by 2.5 [15]. The EES-Q score, ranging from 0 (not at all) to 100 (very severe inconvenience), was calculated by summing the three domain scores and dividing this by 3, indicating equal importance of all 3 health domains. Recently, the EES-Q has specifically been validated for CRS patients with [16] and without surgery [17].

### Statistical analysis

Descriptive statistics were used to summarize patient’s demographics and calculated the percentage of patients in one of the three clinical control groups (controlled, partly or uncontrolled) according to the EPOS criteria. The EES-Q and domain scores were analyzed per clinical control group. Distributions were checked and median and means were used as appropriate. Proportion of patients was compared by Pearson’s chi-squared test. Kruskal–Wallis test was used for comparison of more than two nonparametric data, followed by a post hoc Mann–Whitney U test (including Bonferroni correction). A p value < 0.05 was considered statistically significant. All statistical analyses were performed with IBM SPSS Statistics version 28.0 (SPSS IBM, Inc., Armonk NY, USA).

## Results

### Study population

A total of 123 patients (58.5% male) were included. The mean age was 49.2 ± 13.3 years. Seventy-six (61.8%) were diagnosed with CRSwNP. Twenty-one CRSwNP patients required revision surgery during follow-up (Table 2). Perioperative complications were skull base defect with intact dura (3; 2.4%), an orbital defect (5; 4.1%) and a lachrymal sac defect (1; 0.8%). Postoperatively one patient (0.8%) had a nosebleed that required nasal packing. Over the past 6 months, 4 patients (3.3%) required a single course of rescue treatment (antibiotics and/or prednisolone) in addition to their standard intranasal corticosteroid nasal spray and saline rinsing. Fifty-one patients (41.5%) received more than one course of rescue treatment to manage their symptoms. In 23 patients, these interventions did not provide adequate symptom relief.

**Table 2:** Patient characteristics

Characteristic	*n* (%)
**Sex (male)**	72 (58.5)
**Age, mean (SD) in years**	49.2 (± 13.3)
**Allergic rhinitis**	21 (17.1)
**N-ERD**	18 (14.6)
**Asthma**	33 (26.8)
**Smoking**	29 (23.6)
**Nasal polyps (CRSwNP)**	76 (61.8)
Type of surgery* EES Extended EES	73 (59.3) 50 (40.7)
**Revision surgery during follow-up**	33 (26.8)

Abbreviations: *CRSsNP* Chronic rhinosinusitis without nasal polyps; *CRSwNP* Chronic rhinosinusitis with nasal polyps; *EES* Endoscopic endonasal surgery; *N-ERD* Nonsteroidal anti-inflammatory drug – exacerbated respiratory disease; *SD* Standard deviation

* Type of surgery classified as:

- EES: infundibulotomy, ethmoidectomy, Draf I/IIA, frontal recess surgery

- Extended EES: sphenoidectomy, Draf IIB/III, medial maxillectomy II/III

### Level of disease control

According to the updated EPOS staging system for defining the level of CRS control, symptoms of 31 patients (25.2%) were classified as ‘controlled’, 33 (26.8%) ‘partly controlled’ and 59 (48.0%) ‘uncontrolled’. In our cohort patients with partly controlled CRS presented with 1 symptom (12; 36.4%) or 2 (21; 63,6%) symptoms. Our uncontrolled group included patients with 3 to 6 symptoms. The number of patients with 3, 4, 5, or 6 symptoms was approximately evenly distributed, ranging from 12 to 17 patients (20.3–28.8%).

The effect of different variables on levels of disease control are listed in Table 3. Males and females reported comparable levels of control. Symptoms of 16 (34.0%), 10 (21.3%) and 21 (44.7%) patients with CRSsNP were classified as ‘controlled’, ‘partly controlled’ and ‘uncontrolled’, respectively. Fifteen (19.7%) patients with CRSwNP defined their CRS as ‘controlled’, 23 (30.3%) patients as ‘partly controlled’ and 38 (50.0%) patients as ‘uncontrolled’. Symptoms of 1 (7.6%), 6 (46.2%) and 6 (46.2%) patients with CRSwNP and nonsteroidal anti-inflammatory drug – exacerbated respiratory disease (N-ERD) were classified as ‘controlled’, ‘partly controlled’ and ‘uncontrolled’, respectively. Symptoms of the 5 patients with CRSsNP and N-ERD were partly controlled (2; 40%) or ‘uncontrolled (3; 60%).

**Table 3:** Effect of different variables on updated EPOS level of control *n* (%)

Characteristic (*n*)	Controlled *n* (%)	Partly controlled *n* (%)	Uncontrolled *n* (%)
**All patients** (123)	31 (25.2)	33 (26.8%)	59 (48.0)
**Sex** Male (72) Female (51)	18 (25.0) 13 (25.5)	21 (29.2) 12 (23.5)	33 (45.8) 26 (51.0)
**Nasal polyps** CRSsNP (47) CRSwNP (76)	16 (34.0) 15 (19.7)	10 (21.3) 23 (30.3)	21 (44.7) 38 (50.0)
**Asthma** No asthma (90) Asthma (33)	31 (34.4) none	23 (25.6) 10 (30.3)	36 (40.0) 23 (69.7)
**Smoking** Non-smoker (94) Smoker (29)	25 (26.6) 6 (20.7)	26 (27.7) 7 (24.1)	43 (45.7) 16 (55.2)
**Allergic rhinitis** No (102) Yes (21)	29 (28.4) 2 (9.5)	26 (25.5) 7 (33.3)	47 (46.1) 12 (57.1)
**N-ERD** No (105) Yes (18)	30 (28.6) 1 (5.6)	25 (23.8) 8 (44.4)	50 (47.6) 9 (50.0)
**Type of surgery** EES (73) Extended EES (50)	19 (26.0) 12 (24.0)	21 (28.8) 12 (24.0)	33 (45.2) 26 (52.0)
**Revision surgery during follow-up** No (90) Yes (33)	29 (32.2) 2 (6.1)	24 (26.7) 9 (27.3)	37 (41.1) 22 (66.7)
***Pearson’s Chi-square tests p-value***			
**Sex**	0.369	0.117	0.362
**Nasal polyps**	0.857	0.024	0.027
**Asthma**	n/a	0.024	0.091
**Smoking**	0.001	0.001	< 0.001
**Allergic rhinitis**	n/a	0.001	< 0.001
**N-ERD**	n/a	0.003	< 0.001
**Type of surgery**	0.209	0.117	0.362
**Revision surgery during follow-up**	n/a	0.009	0.051

Abbreviations: *CRSsNP* Chronic rhinosinusitis without nasal polyps; *CRSwNP* Chronic rhinosinusitis with nasal polyps; *EES* Endoscopic endonasal surgery; *N-ERD* Nonsteroidal anti-inflammatory drug – exacerbated respiratory disease

Significant association was found between the level of disease control and several variables (Table 3). For patients with a controlled level of CRS non-smoking was a positive contributor (*p* = 0.001). A partly controlled level of CRS was positively associated with nasal polyps (*p* = 0.024), asthma (*p* = 0.024), non-smoking (*p* = 0.001), allergic rhinitis (*p* = 0.001), N-ERD (*p* = 0.003) and revision surgery (p = 0.009). An uncontrolled level of CRS was positively associated with nasal polyps (*p* = 0.027), smoking (*p* < 0.001), allergic rhinitis (*p* < 0.001) and N-ERD (*p* = < 0.001). Borderline significance was found for revision surgery (*p* = 0.051) in this group

### EES-Q (domain) scores per level of control

**Fig. 1:**
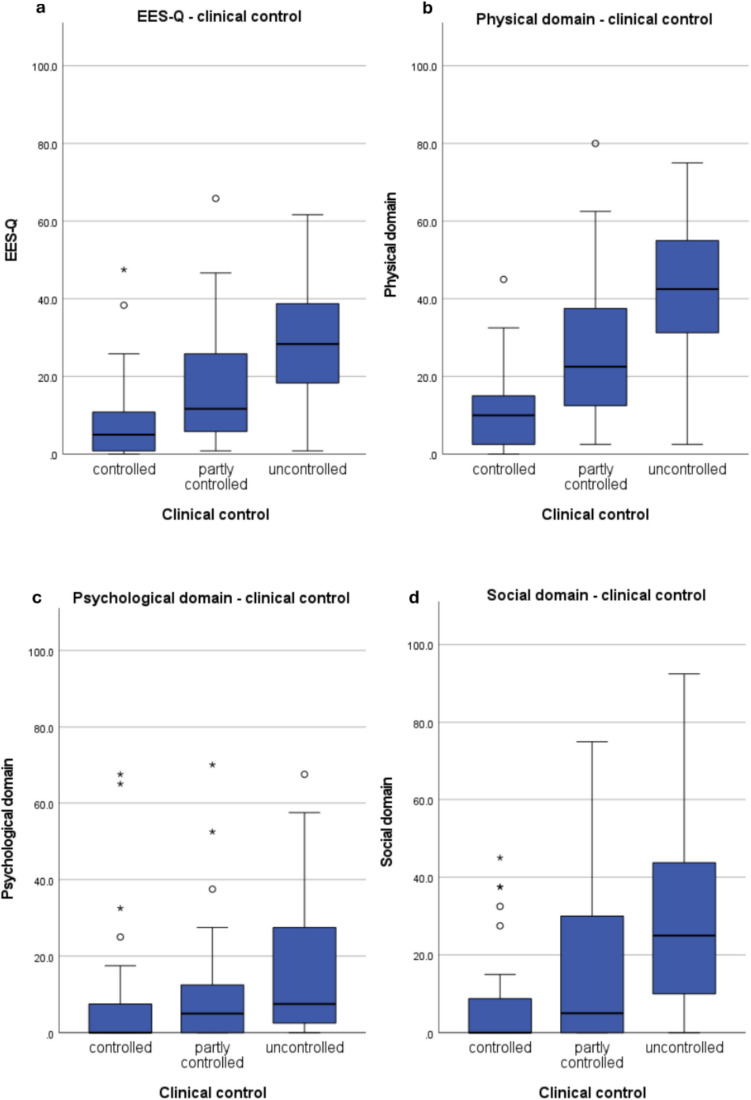
Median (IQR) EES-Q and domain scores per clinical control group (◦ = mild outlier, * = extreme outlier)

The median (IQR) EES-Q score reported by all patients was 19.17 (6.67–31.67). The median score of the physical domain was 27.50 (15.00–47.50). Within the physiological and social domains median scores were 5.00 (0.00–20.00) and 12.50 (0.00–37.50), respectively. For reference, mean scores were as follows: 20.96 (EES-Q score), 30.24 (physical domain), 12.46 (psychological domain) and 20.18 (social domain). When patients were classified in the 3 groups (controlled, partly controlled and uncontrolled CRS) significant differences were seen between the EES-Q score and all 3 domain scores (all *p* < 0.01) (Table 4). Post hoc analysis shows that EES-Q and physical and social domain scores significantly differ between all 3 clinical control groups (*p* < 0.05). The psychological domain score only significantly differed between controlled vs. uncontrolled CRS (*p* = 0.001). Figure 1 shows the median (IQR) per domain per level of control.

**Table 4:** Comparison of EES-Q (domain) scores by disease control assessment using the updated EPOS 2020 staging system > 5 years after EES

3-way Kruskal–Wallis analysis				
	EPOS disease control assessment, Median (IQR)			
	**Controlled** ***n*** **= 31**	**Partly controlled** ***n*** **= 33**	**Uncontrolled** ***n*** **= 59**	***p–value***
**EES-Q score**	5.00 (0.83–10.83)	11.67 (5.00–28.33)	28.33 (17.50–39.17)	< 0.001
**Physical domain**	10.00 (2.50–15.00)	22.500 (12.50–37.50)	42.50 (30.00–55.00)	< 0.001
**Psychological domain**	0.00 (0.00—7.50)	5.00 (0.00–16.25)	7.50 (2.50–27.50)	0.004
**Social domain**	0.00 (0.00–10.00)	5.00 (0.00–31.25)	25.00 (10.00–45.00)	< 0.001
Post hoc Mann–Whitney U analysis				
	**Controlled vs. partly controlled**	**Controlled vs. uncontrolled**	**Partly controlled vs. uncontrolled**	
**EES-Q score**	0.002	< 0.001	< 0.001	
**Physical domain**	< 0.001	< 0.001	< 0.001	
**Psychological domain**	0.162	0.001	0.061	
**Social domain**	0.016	< 0.001	0.005	

Abbreviations: *EES-Q* Endoscopic Endonasal Sinus and Skull Base Surgery Questionnaire; *EPOS* European Position Paper on Rhinosinusitis and Nasal Polyps; *IQR* Interquartile range

## Discussion

This is the first study evaluating the updated EPOS criteria by comparing them with the EES-Q. We aim to achieve more insight into the concept of clinical control in CRS patients more than five years after EES. We found that symptoms of 25% of patients were classified as ‘controlled,’ 27% as ‘partly controlled,’ and 48% as ‘uncontrolled,’ according to the updated EPOS staging system. This is consistent with the findings of Van der Veen et al., who – based on the 2012 criteria—reported 20%, 37%, and 44% for controlled, partly controlled, and uncontrolled levels of CRS, respectively [11]. However, the EPOS criteria have been updated since, as they were thought to overestimate the rate of uncontrolled CRS. More recent studies using the updated EPOS grading system reported varying results (controlled: 4.5–31.9%, partly controlled: 22.6–44.2% and uncontrolled: 23.9–72.9%) [7, 18]. Although, our results fell within this range; the variability was too broad to clearly differentiate between the different levels of control. Even though patients with uncontrolled or partially controlled CRS might still have benefited from surgery, this outcome did not meet our clinical expectations. In our cohort, N-ERD, allergic rhinitis, revision surgery, smoking, asthma, and nasal polyps were all associated with partly controlled or uncontrolled disease. This was expected, as these factors have been previously linked to poorer HRQoL measures [2, 7, 11, 19–23]. This emphasizes the importance of adequate counseling.

Today, it is well known that the patient’s perspective of HRQoL should be taken into account, as emphasized by a recent study reporting a discordance of disease control between patients and the EPOS guidelines [18]. While the EPOS differentiated between levels of disease control, the EES-Q provided important insights into the multidimensional nature of CRS. Although we found significant differences within the domains, the dispersion was too large to allow for a reliable classification. EES-Q scores showed little dispersion for the controlled CRS group, implicating a reasonably good cut-off value. However, it was not possible to distinguish between the partly controlled and uncontrolled group because of the large dispersion. Interestingly, comparing these results with the EES-Q results of our healthy control group of a previous study showed good comparison with the partly controlled patients [16].

As expected, the physical domain played the most significant role in disease control [5, 6]. The controlled group showed a relatively small dispersion compared with the partly controlled and uncontrolled groups. Scores of our healthy control group fell between those of controlled and partly controlled CRS [16], emphasizing the need to critically evaluate the current cut-off scores of the EPOS criteria. Social and occupational activities are crucial to CRS patients [9, 24, 25]. The social domain demonstrated some distinction between the clinical control groups but also showed excessive dispersion across the scale. Highlighting individual differences and the importance of this domain. Unfortunately, previous studies used general health questionnaires to address this, rather than evaluating the social domain separately [5, 11, 26]. The scores of controlled and partly controlled patients closely resembled those of healthy controls [16]. Raising – again- concerns about the cut-off thresholds of the EPOS criteria. The psychological domain showed a considerable dispersion, likely due to its subjective nature. Scores of healthy individuals were comparable with those of the partly controlled group [16]. Given that CRS is associated with psychological disorders [7, 8, 27] and that poor mental health increases healthcare-seeking behavior [28] and indirect costs [3], this domain requires a more refined approach in clinical control assessments.

### Limitations

Patients were not asked to rate their own CRS control status, which could have provided valuable additional insights. Despite the fairly large number of patients included in the study, the number of patients per category was relatively small. Even though it was beyond the scope of the current study, it would be interesting to prospectively measure changes in clinical control over time. Finally, this study classified patients based on phenotypic aspects of CRS. We did not take the newly suggested classification by endotype dominance into account [2]. With the growing evidence that inflammatory endotypes, particularly type 2, play a crucial role in CRS pathophysiology and treatment response, future research should incorporate endotype classification. This study was conducted at a tertiary referral centre, potentially causing a selection bias towards more severe CRS cases. Nevertheless, the validation study of the EES-Q [17] previously demonstrated no significant differences between CRS patients from secondary or tertiary referral hospitals.

## Conclusion

Even based on the updated EPOS criteria, the percentage of patients with a controlled level of CRS seems to be underestimated. This may be attributed to the multidimensional nature of CRS, which is not fully captured by the EPOS criteria. Our findings highlight the importance of considering not only the physical complaints, but also the social and psychological domains. Despite the significant differences observed within these domains, the wide dispersion of results hinders effective and informed classification indicating a need for further refinement. Additionally, the similarity between scores of partly controlled patients and healthy individuals raises concerns about the current cut-off thresholds. Concluding, improving on the clinical criteria potentially improves patient classification and therefore more patient centered treatment strategies.

## Supplementary Information

Below is the link to the electronic supplementary material.

Supplementary file1 (DOCX 23.8 KB)

## Data Availability

The data supporting the findings of this study are available upon request from the authors. The data are not publicly available due to privacy or ethical restrictions.
