# Supplementary material for: Long-term clinical control in chronic rhinosinusitis: Outcomes more than five years after surgery
Source: Eur Arch Otorhinolaryngol. 2025 Aug 9;282(9):4661–8. doi: 10.1007/s00405-025-09529-z (PMC12423212; doi:10.1007/s00405-025-09529-z)
Supplement: Supplementary file 1 — Supplementary file1 (DOCX 23.8 KB) [file 405_2025_9529_MOESM1_ESM.docx]

|  | **Not at all** | **Mildly** | **Moderately** | **Severely** | **Very severely** |
| --- | --- | --- | --- | --- | --- |
| 1. Blocked nose | □ | □ | □ | □ | □ |
| 2. Difficulty breathing through nose | □ | □ | □ | □ | □ |
| 3. Dry mouth | □ | □ | □ | □ | □ |
| 4. Need to blow nose | □ | □ | □ | □ | □ |
| 5. Reduced sense of smell | □ | □ | □ | □ | □ |
| 6. Reduced sense of taste | □ | □ | □ | □ | □ |
| 7. Facial pressure | □ | □ | □ | □ | □ |
| 8. Fitful sleep | □ | □ | □ | □ | □ |
| 9. Tired | □ | □ | □ | □ | □ |
| 10. Waking up tired | □ | □ | □ | □ | □ |
| 11. Feeling depressed | □ | □ | □ | □ | □ |
| 12. Sad | □ | □ | □ | □ | □ |
| 13. Feeling down | □ | □ | □ | □ | □ |
| 14. Distressed | □ | □ | □ | □ | □ |
| 15. Aggravated | □ | □ | □ | □ | □ |
| 16. Frustrated | □ | □ | □ | □ | □ |
| 17. Stressed | □ | □ | □ | □ | □ |
| 18. Tense | □ | □ | □ | □ | □ |
| 19. Worried | □ | □ | □ | □ | □ |
| 20. Discouraged | □ | □ | □ | □ | □ |
| 21. Work / study | □ | □ | □ | □ | □ |
| 22. Leisure time activities | □ | □ | □ | □ | □ |
| 23. Hobbies | □ | □ | □ | □ | □ |
| 24. Indoor activities at home | □ | □ | □ | □ | □ |
| 25. Outdoor activities at home | □ | □ | □ | □ | □ |
| 26. Going out for a visit | □ | □ | □ | □ | □ |
| 27. Shopping | □ | □ | □ | □ | □ |
| 28. Daily activities | □ | □ | □ | □ | □ |
| 29. Engaging in outdoor activities | □ | □ | □ | □ | □ |
| 30. Physical exertion | □ | □ | □ | □ | □ |

This questionnaire contains complaints that can be the result of a disorder of the paranasal sinuses. For each complaint, indicate whether you have experienced it **in the past 2 weeks** and if so, how severe it was.

Activities are also mentioned in which you may be hindered in performing as a result of your complaints. For each activity, indicate to what extent you have been **hindered** in the past 2 weeks.
